# Designing, Implementing and Optimising a Capacity‑Building Model for Infectious Disease Modelling in India

**DOI:** 10.5334/aogh.4606

**Published:** 2024-12-30

**Authors:** Jaya Prasad Tripathy, PVM Lakshmi, Tanu Anand, Pradeep R Deshmukh

**Affiliations:** 1 Department of Community Medicine, All India Institute of Medical Sciences, Nagpur, India; 2 Department of Community Medicine and School of Public Health, Post Graduate Institute of Medical Education and Research, Chandigarh, India; 3 Scientist‑E, Division of Development Research, Indian Council of Medical Research, New Delhi, India

**Keywords:** infectious disease modelling, course development, capacity building, training, mathematical modelling

## Abstract

*Background:* Mathematical models are not integrated into the policy‑making process in low‑ and middle‑income countries, including India, primarily due to limited capacity in building mathematical models, lack of trust in the model findings and the reluctance of policy‑makers to apply the model findings to formulate policies. There is a perceived need to create a critical mass of trained infectious disease experts and modelers within the public health and clinical domain. Thus, with the support of the Department of Health Research (DHR), we developed a 3‑month post‑graduate (PG) certificate course on infectious disease modelling, the first of such a course in India. The first cycle of the course was delivered during July to September 2024, which produced the first cohort of 20 infectious disease modellers in the country.

*Methods:* This paper describes the structure, content and key components of the first course along with the experiences, strengths, challenges and way forward from the participants’ perspective using a mixed methods approach.

*Findings:* Most of the participants felt that the learning objectives were clear (*n* = 18, 90%), course content was well organised and delivered (*n* = 19, 95%) and the course structure allowed all participants to fully participate (*n* = 19, 95%) in the learning process. The strengths of the course were: hybrid mode of delivery, well‑designed course content, theory lectures followed by practical sessions, regular trainer–trainee communication, interactive discussion forums and the 3‑day contact workshop. The key challenges were non‑availability of recorded videos, evening timings of the sessions and difficulty of some topics.

*Conclusions:* The challenges and recommendations will feed into the subsequent course cycles. Future courses are planned to be hosted on an online platform to facilitate participant completion of the course at their own pace. More collaboration with various stakeholders, nationally and internationally, will be sought to improve the content, delivery and robustness of the program.

## Introduction

Infectious diseases remain a leading cause of morbidity and mortality worldwide, estimated to cause more than 10% of deaths and 28% of disability‑adjusted life‑years (DALYs) attributed to all causes in 2019, with human immunodeficiency virus (HIV), tuberculosis and malaria being the key contributors [1]. Outbreaks of Ebola and coronavirus disease 2019 (COVID‑19) in recent years have led to unprecedented numbers of deaths and cases. New pathogens continue to emerge in animal and human populations, as demonstrated by the emergence of severe acute respiratory syndrome (SARS) in 2003, highly pathogenic avian influenza in poultry and humans in 2004/2005, swine flu in 2009, Middle East respiratory syndrome coronavirus (MERS‑CoV) in 2013, Zika in 2016, severe acute respiratory syndrome coronavirus 2 (SARS‑CoV‑2) in 2019 and recently, the monkeypox virus in 2022 and 2024 [2].

Mathematical models are being increasingly used to understand the transmission of infections and to evaluate the potential impact of control measures or interventions in reducing morbidity and mortality. Mathematical modelling underpinned most of the critical decisions made by the UK government during the COVID‑19 pandemic, including the decision to implement a nationwide lockdown in March 2020, lay down a road map for release from lockdown in February 2021 and implementation of public health interventions in December 2021 during the omicron wave [3]. In the USA, modelling projections for different COVID‑19 scenarios by the Institute for Health Metrics and Evaluation COVID‑19 Forecasting Team also informed crucial policy decisions [4]. The use of mathematical disease models in public health policy is well adapted to the decision‑making process for epidemic and endemic diseases in high‑income countries [5]. Even organisations such as the World Health Organization (WHO) and the Joint United Nations Programme on HIV/AIDS (UNAIDS) have relied on findings from mathematical modelling studies to make crucial choices around selection of the intervention and vaccination strategies for diseases such as influenza, Ebola, HIV and COVID‑19 [6].

It is encouraging to see African countries demonstrating global leadership in infectious disease mathematical modelling through successful north–south collaborations. Organisations such as South Africa’s Modelling and Simulation Hub Africa (MASHA), the South African Centre of Excellence in Epidemiological Modelling and Analysis (SACEMA) and the Centre for Infectious Disease and Epidemiology Research (CIDER) have played a key role in increasing outputs related to disease modelling studies by African researchers. They have also supported national governments in making model‑informed evidence‑based policies [7]. Training and mentorship have been identified as key approaches to strengthening mathematical modelling capacity in Africa and elsewhere [8]. A deliverable‑driven mentor‑led learning‑by‑doing model of capacity building for policy‑makers and public health professionals in Africa was an effective training model for building the capacity in mathematical modelling of diseases [9].

In India, these disease models are not fully integrated into the policy‑making process, primarily due to limited capacity in building mathematical models, lack of trust in the findings given the many assumptions and data limitations and the reluctance of policy‑makers to apply the model findings to formulate policies [10]. Much of this lack of trust or reluctance in adopting the model findings stems from the lack of knowledge about how these models are conceived, constructed and calibrated. During the COVID‑19 pandemic, although several mathematical models were proposed to understand the evolving disease in India, they did not feed into the process of decision‑making [11]. The possible reasons could be: (i) large variations in the model predictions and assumptions, breeding mistrust in the model results; (ii) criticisms around use of simple mathematical models to describe complex processes; (iii) use of models to describe real‑life epidemic scenarios being relatively new; and (iv) lack of knowledge about what goes on in the building of such models.

Thus, there was a perceived need to create a critical mass of trained infectious disease experts and modellers within the public health and clinical domain so that they could work closely and support the district, state and national governments to understand disease spread and transmission dynamics during epidemics and support model‑informed decision‑making. This need was more felt during the recent COVID‑19 pandemic.

Previous training models in India were short‑term courses predominantly based on didactic teaching ranging from 2 to 5 days and covering only the basics of infectious disease modelling without being deliverable‑driven and devoid of long‑term mentorship. Following the COVID‑19 pandemic, several disease modelling experts have come together to form groups such as the National Disease Modelling Consortium and the Indian Scientists’ Response to CoViD‑19 (ISRC) to develop India‑specific disease models to aid national policy‑makers make informed decisions and improve disease control and elimination efforts [12, 13]. However, training and mentorship have never been at the forefront of their agenda. Recognising this gap in training, the Department of Health Research (DHR), which is the Government of India body for research in India, released a call for applications under the Human Resource Development Scheme to design long‑term capacity‑building programs in key priority areas, including infectious disease modelling. In response to the call, we proposed a 3‑month post‑graduate (PG) certificate course in infectious disease modelling in hybrid mode to the DHR in order to build a team of infectious disease modellers who can prove to be a great asset in tackling future pandemics and emerging threats. Following sanction by the DHR, we developed the course structure and curriculum and delivered the first cycle of the course during July to September 2024, producing the first cohort of 20 infectious disease modeilers in the country. The course curriculum was guided by Kolb’s experiential learning theory, which is an andragogical approach to learning focussing on real‑world experiences and practical applications [14]. This was the first such course on infectious disease modelling in India. The structure, content and key components of the first course, along with the strengths, challenges and way forward from the participants’ and facilitators’ perspective, are discussed in this paper below.

## Methods

**Study design:** This was a mixed‑methods approach to evaluate a capacity‑building program on infectious disease modelling.

**Study setting:** We describe the design and development of the capacity‑building model below.

### Course development

This is a learning‑by‑doing model of andragogical training conceived and designed by faculty from the All India Institute of Medical Sciences (AIIMS) Nagpur, Post Graduate Institute of Medical Education and Research (PGIMER), Chandigarh, and the Indian Council of Medical Research (ICMR), New Delhi, India. The faculty are experienced epidemiologists with special interest and expertise in infectious disease epidemiology and modelling. The development of the curriculum was guided by Kolb’s experiential learning theory [14]. There are four stages, which begin with having a concrete learning experience, followed by reflective observation and abstract conceptualisation, and ending with them actively experimenting with the knowledge they gained.

We delivered concrete learning experiences through a series of online lectures, recorded videos, self‑reading materials and practical exercises with reflections after each practical exercise, open discussion forums and Q&As. We built in opportunities for the participants to conceptualise the process through biweekly assignments which were reviewed, and in‑depth inputs were provided. Every participant was supposed to submit a project by the 11th week involving designing and optimising a specific infectious disease model by applying the knowledge learnt during the course. This provided the participants with the chance to experiment with their newly gained insights in a practice situation in a highly mentored environment.

### Course goal

The overall goal of this initiative was to strengthen the capacity in mathematical disease modelling to enhance their use in decision‑making and effective communication of modelling outputs to policy‑makers in India.

### Course objectives

1. Understand the basic concepts of infectious disease dynamics and mathematical modelling.
2. Construct valid mathematical models capturing the natural history of a disease.
3. Design, optimise and build a disease model for different diseases and intervention scenarios using software.
4. Understand the strengths and limitations of a model in varying research and policy contexts.
5. Critically assess disease models and make decisions on the basis of model findings.

### Course details

#### Selection of participants

The course participants included regular faculty/scientists/PhD students/post‑doctoral students from medical colleges and research institutes, biostatisticians, veterinarians, public health and clinical researchers from government institutes, non‑governmental organisations (NGOs) or other organisations from India and policy‑makers and disease control professionals with interest and background in infectious disease modelling. Specialist mathematical training was not a prerequisite. However, some familiarity with spreadsheet packages (Microsoft Excel) was desirable.

Selection of participants was competitive and individuals with prior experience in the infectious disease domain and those who committed to taking this capacity‑building initiative forward in their respective institutions were preferred. The applications were scored using a structured scoring sheet. The criteria used to score were: highest educational qualification, graduation marks, work experience in the field of infectious diseases, publications and research projects in the domain of infectious diseases and any fellowship/diploma/PG or any equivalent course in infectious diseases. No course fee was charged to the participants.

#### Who is a successful participant?

A participant successfully completes the course and gets the certificate if they fulfil all the following criteria:

- Attends at least 75% of the online sessions
- Submits all four assignments and the project work to the satisfaction of the facilitators before the deadline
- Attends all the offline contact sessions
- Completes the final exit examination scoring at least 50% marks

#### Capacity‑Building model

**Figure 1:**
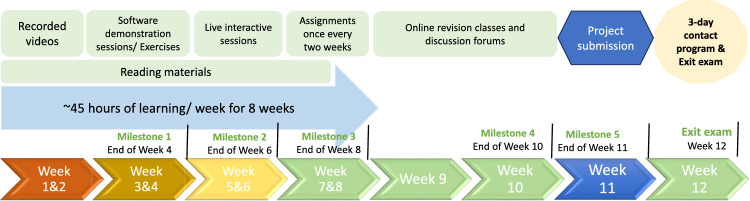
Details about the course structure, modes of delivery, milestones, deliverables and assessment methods.

The details of the course structure and the delivery of the course are described in Table 1 and Figure 1. Supplementary appendix 1 shows the details of the curriculum including the week‑wise course content and the teaching learning methods. The course was delivered via a six‑step process:

1. **8‑week online course:** This was delivered through live online video lectures, online software demonstrations and exercises, once weekly live discussion forums, bi‑weekly assignments and reading materials. The total duration of teaching was around 45 hours per week.
2. **Bi‑weekly assignments:** After the completion of every 2 weeks, assignments were given. All four assignments had to be submitted within the specified deadlines. (Milestones 1‑4).
3. **Project work:** A project‑based assignment was given wherein they will be practically applying the principles learnt. The final project report had to be submitted before the completion of the 10th week of the course. The format in which the project report is to be submitted is given in Supplementary appendix 2 (Milestone 5).
4. **Online revision** and discussion classes in Week 11.
5. **3‑day contact programme:** A 3‑day contact programme was held in the 12th week of the course to discuss and revise the key concepts, clarify doubts and give them a mentored hands‑on practice on the key exercises.
6. **Exit examination:** The contact programme was followed by an exit examination the very next day.

**Table 1:** Details about the course structure and delivery.

DOMAIN	COURSE DETAILS
Approach	Deliverable‑driven hands‑on approach to training with intensive mentorship during practicum and in‑person sessions
Mode of delivery	Hybrid mode (online video lectures, discussions and demonstrations, offline contact programme and exit examination)
Target trainees	Public health professionals, medical college faculty, biostatisticians, microbiologists and scientists working in the domain of infectious diseases
Duration of the course	12 weeks (including 8 weeks of online training, followed by assignments and project submission, face‑to‑face contact session and examination)
Deliverables	Four assignments and project work
Course advertisement	Advertised within the priority organisations, professional networks and on social media such as LinkedIn, Facebook and Twitter
Trainee selection and number	A total of 24 participants were selected on the basis of their previous clinical, programmatic or research experience in the domain of infectious diseases out of 224 applications received.
Course format	Live online video lectures, online hands‑on practical exercises and software demonstrations, live discussion forums and Q&A, case studies, journal clubs, bi‑weekly assignments and project submission, 3‑day contact session followed by final exit examination
Course fee	No course fee was charged to the participants. However, the participants had to bear their cost of travel, accommodation and other expenses during the face‑to‑face contact session and the exit examination
Assessment	Formative assessment: Four assignments (25 marks each) Summative assessment (100 marks): End‑of‑course exit examination (75 marks) Theory (50 marks) Practical (25 marks) End‑of‑course project submission (25 marks)
Course feedback and evaluation	Participants evaluated the structure and content of the training at the end of each week of training through formal and informal feedback mechanisms to inform subsequent sessions. In addition, trainees provided overall evaluation of the course at the end of the training, including training logistics.

Table 2 provides details about the week‑wise course topics and milestones.

**Table 2:** Details about the week‑wise course topics and milestones.

WEEK	TOPIC	MILESTONES
1	Introduction to infectious disease epidemiology	–
2	Setting up compartmental models	–
3	Excel and math basics	–
4	Introducing demography and age structure into the model	Milestone 1: Submission of the first assignment by week 4
5	*R*_0_, herd immunity and effect of vaccination	–
6	Effect of control measures or interventions	Milestone 2: Submission of the second assignment by week 6
7	Stochastic models and incorporating non‑random mixing	–
8	Modelling other diseases: HIV, TB, malaria, COVID‑19	Milestone 3: Submission of the third assignment by week 8
9	Online revision and discussion forum	–
10	Online revision and discussion forum	Milestone 4: Submission of the third assignment by week 10
11	Online revision and discussion forum	Milestone 5: Submission of the project work by week 11
12	Contact session and exit examination	Passing the exit examination

HIV, human immunodeficiency virus; TB, tuberculosis; *R*_0_, basic reproduction number; COVID‑19, coronavirus disease 2019.

**Study participants:** The study population included all participants (*n* = 20) who completed all milestones (required online and offline session attendance, submission of assignments and project work) and were eligible for the final exit examination and the course facilitators.

### Data collection and variables

Self‑administered, semi‑structured questionnaires were emailed to the course participants (*n* = 20) via Google form after completion of the face‑to‑face offline sessions. Anonymous feedback was collected to get appropriate responses without any desirability bias. Identifying information and email IDs were not collected. The questionnaire included closed‑ended quantitative and open‑ended qualitative variables. The quantitative variables included feedback on the overall course content, learning objectives, balance between theory and hands‑on, delivery of the course, contribution of the course towards learning and skill and responsiveness of the facilitators. A five‑point Likert scale was used to record the responses. The qualitative variables included open‑ended questions assessing strengths (what worked well?), weaknesses (what did not work well?) and suggestions to improve the delivery of the course in subsequent cycles. Facilitators’ feedback regarding the strengths and weaknesses of the course and suggestions for improvement in the subsequent cycles was also taken in a meeting of the facilitators after the course.

### Data analysis

The data (both quantitative and qualitative) were captured in MS Excel format. Quantitative variables were summarised using proportions. The responses ‘very good’ and ‘excellent’, as well as ‘agree’ and ‘strongly agree’, were combined to form a single category.

Manual descriptive content analysis of the textual responses to the open‑ended questions was carried out by two authors (J.P.T. and P.D.), who are experienced in qualitative research. Themes were generated in consensus using standard procedures by a deductive approach [15]. Any disagreement between the two authors was resolved by mutual discussion. The participants were contacted again by email or telephone in case any clarification was required. Statements in italics represent direct quotes from the participants.

### Ethics approval

Obtaining feedback from the participants was performed as part of routine evaluation of the training mandated by the funding agency. Thus, approval from the ethics committee was not deemed necessary. Feedback was completely anonymous and participants were free not to respond to the questionnaires.

## Results

### Quantitative findings

#### Background of the selected participants

Out of 224 applicants, a total of 24 participants were selected for the first cohort. The mean age of the participants was 37.7 years (standard deviation 4.9), ranging from 29 to 48 years. About 42% (*n* = 10) were female. Most of them belonged to a public health background (*n* = 16, 66.6%), followed by biostatisticians (*n* = 4, 16.7%) and microbiologists (*n* = 4, 16.7%). Those from a public health background came from diverse domains including medical college faculty, scientists from ICMR institutes, junior and senior residents, national consultants working with the World Health Organization, state‑level public health administrators, etc. Only one of them (4.2%) had attended a course on mathematical modelling of infectious disease before. Of the 24 selected participants, 20 (83.3%) successfully completed the course; 1 dropped out of the course very early in the first month and the remaining 3 could not attend the contact workshop due to other competing personal or professional commitments, and thus were ineligible for the final exit examination.

Out of 20 participants, about three‑fourths (*n* = 15, 75%) felt that the contribution of the course towards enhancing their knowledge was ‘very good’ or ‘excellent’. Most of them felt (‘agree’ or ‘strongly agree’) that the learning objectives were clear (*n* = 18, 90%), course content was well organised and delivered (*n* = 19, 95%) and the course structure allowed all participants to fully participate (*n* = 19, 95%) in the learning process. They believed that the course instructors were effective teachers (*n* = 20, 100%), stimulated student interest (*n* = 19, 95%) and were available and helpful (*n* = 20, 100%). All the participants (*n* = 20, 100%) found this course useful and would recommend it to their colleagues enthusiastically.

### Qualitative findings

The following broad themes emerged: strengths of the course, challenges and way forward from a participants’ perspective.

#### Strengths of the course

**COURSE CONTENT: THEORY FOLLOWED BY HANDS‑ON SESSIONS**

Most of the participants felt that practical exercises after the basic theory lecture were extremely helpful in understanding the concepts and their applications.

*‘Theory followed by practical sessions were extremely helpful and easy to follow, interactive’.*

*‘Practical exercises after each theory was the highlight of the course’.*

The practical hands‑on sessions and the discussions following that were especially useful in the understanding of complex concepts. The participants suggested more such exercises and discussions in subsequent courses.

*‘The most valuable aspect of the online course was the practical sessions. They were truly outstanding and greatly enhanced my understanding of the subject matter. The clear explanations, examples and hands‑on approach made complex concepts much easier to grasp’.*

### Interactive contact sessions

Most of the participants found the 3‑day contact workshop very useful as there were many practical hands‑on activities, small group activities, interactive discussions and less theory lectures, which was not possible during the online sessions. It also helped them revise and consolidate the concepts learnt earlier, especially the complex topics during the latter half of the course. Some of them even said that some complex topics such as modelling HIV/sexually transmitted infections (STIs) and mixing of populations were difficult to follow online, but the in‑person sessions were useful in clarifying them.

*‘Yes definitely. This revision was a must. Since week 5 the content became very hifi which was made clear during the offline session’.*

*‘Lot of practical works and less of theory sessions ‑ was a good way to engage and empower participants*’.

*‘The interactive nature of the sessions, the attentive nature of instructors to the problems faced by individual participants. All teachers were ready to take questions. We got enough time to discuss theory and exercises’.*

### Support from trainees’ institutions

The participants reported that support from trainees’ institutions to pursue the course and attend all online and offline sessions was important. The application process mandatorily required applicants to submit a letter of support from their employers, which meant that they could focus on the course and devote sufficient time without having to worry about their full‑time work commitments.

The facilitators reported that trainer–trainee communication through various forums and the trainee’s commitment were critical to the success of this cohort.

### Trainer–Trainee communication

A key feature of this program critical for trainees’ success was the regular communication between trainees and trainers through regular online sessions, online discussion forums, Q&As and the practical hands‑on sessions which provided trainees the space to implement the concepts learned and to receive feedback. We created a WhatsApp group to facilitate easier communication between trainers and trainees as well as knowledge sharing and networking among trainees. Additionally, during in‑person sessions, trainers were available for discussions at the end of each training day.

### Trainees’ commitment

Another facilitator of success was the trainees’ commitment, demonstrated by completing assignments and project work on time and attending evening online sessions regularly amidst competing commitments from their full‑time work.

#### Challenges and way forward

**SCANTY COVERAGE OF THE BASICS OF INFECTIOUS DISEASES**

The participants also gave useful feedback on the challenges they encountered during the course. A participant from a non‑medical background commented that the basics of common infectious diseases and their natural history should be discussed thoroughly. More real‑life examples of mathematical models from the literature for common diseases should be discussed.

*‘I feel that the basics of infectious diseases should be discussed more thoroughly. Emphasis should be more on building a conceptual understanding. More real‑life examples about mathematical modelling should be discussed especially for more common diseases like measles, mumps, influenza, rubella etc.’*

‘For *non‑medical candidates, extra sessions may be provided on basics of disease epidemiology*’.

**LESS TIME FOR DISEASE‑SPECIFIC MODELLING**

Some of the participants felt that the disease‑specific modelling topics such as TB, HIV and sexually transmitted infections (STIs) were difficult to grasp and needed more time.

*‘Disease‑specific modelling needs more classes with discussion of different models, feel like rushed, found bit difficult’.*

**TIMING OF ONLINE SESSIONS AND NON‑AVAILABILITY OF RECORDED VIDEOS**

Timing of the evening online sessions and non‑availability of recorded videos were also reported by some participants as a challenge.

*‘Evening timing of lectures could be scheduled from 6:30 to 7:30pm. At times faced difficulty in taking call at 6pm’.*

Table 3 presents the challenges and recommendations given by the participants and suggested a plan of action for future courses.

**Table 3:** Challenges and recommendations for future Infectious Disease Modelling courses.

TOPIC	CHALLENGES/SUGGESTIONS	ACTION PLANNED FOR SUBSEQUENT COURSES
Pre‑reading materials	Pre‑reading materials before some of the sessions	Pre‑reading materials will be collected and circulated before the sessions
Timing of the sessions	Attending evening online lectures was challenging for the working professionals	In the subsequent courses, a web‑based course portal will be designed wherein lecture videos and other resource materials will be uploaded and the participants can complete the course at their own pace
Recorded lecture videos	Non‑availability of recorded lecture videos	A web‑based course portal will be designed wherein recorded lecture videos will be uploaded
Focus on basics of infectious diseases	Basics of infectious diseases should be discussed more thoroughly, especially for the non‑medical participants	More lectures and reading materials will be included to cover the basics of common infectious diseases in the first 2 weeks
Difficult topics	Disease‑specific modelling topics such as HIV, TB and STIs needs more time and discussion	We will revisit the lectures and add some case studies and exercises to elaborate on disease‑specific modelling
Course content	More exercises, quizzes, case scenarios, journal clubs	We will add more exercises for practice, quizzes at the end of every 2 weeks and some real case scenarios for discussions

HIV, human immunodeficiency virus; TB, tuberculosis; STIs, sexually transmitted infections.

## Discussion

This is the first study using a mixed methods approach to evaluate learner’s perceptions of an innovative 3‑month hybrid training program in infectious disease modelling targeting mid‑career professionals in India. This paper describes the structure, curriculum and delivery of the course and also highlights the strengths and challenges in training the first cohort of disease modellers along with recommendations for the subsequent cohort.

Some of the participants who belonged to the non‑medical background suggested that more focus should be on the basics of infectious disease epidemiology, disease transmission, natural history of diseases and their prevention and management. Accordingly, we plan to include more recorded lectures and discussions on those topics, including the clinical aspects of these diseases in the first 2 weeks, so that everyone is on the same page irrespective of their educational background before we move into the modelling of these diseases.

The working professionals reported that attending evening online lectures around 6 PM, 5 days a week was challenging, as it was their commuting time. Non‑availability of recorded videos was also reported by many as it did not allow them to revise the concepts and make up for their missed classes, if any. To offset these challenges, we are designing a web‑based course portal for the subsequent courses wherein lecture videos and other resource materials will be uploaded and the participants can complete the course at their own pace.

The number of applications (*n* = 224) far exceeded the number anticipated by the team, which demonstrated the demand of the course. These applications were processed objectively using a structured scoring sheet which took longer than planned and required substantial effort from the trainers. Additional personnel support for program coordination might have helped.

Further, given that most of the content was developed originally for this course, the time and effort required to prepare the course content was substantial. However, we can leverage the course materials of the first cohort for future courses, although the materials need to be tailored to specific trainee populations.

A major limitation in this study was the self‑reporting of strengths and weaknesses by the authors of the papers, who were also the respondents in this study. Thus, responder bias cannot be ruled out. However, responses from the study participants were completely anonymised to minimise social desirability bias. In addition, responses to the open‑ended questions were obtained online, leaving no scope for probes and further in‑depth exploration, thus affecting the richness of the qualitative data.

## Conclusions

This is the first structured 3‑month PG certificate course in India attempting to build the capacity of researchers in the field of infectious disease modelling and its applications. The first cycle of the course yielded 20 trained infectious disease modelers in the country. There were some challenges and recommendations from the first cycle which will feed into the subsequent course cycles. Future courses are planned to be hosted on an online platform to facilitate the completion of the course at the participants’ own pace and be able to access the course materials and online videos at any time. More collaboration with various stakeholders, nationally and internationally, will be sought to improve the content, delivery and robustness of the program.

## Additional File

The additional file for this article can be found as follows:

Supplementary appendix 1. Details about the week‑wise course topics, content, teaching–learning methods and milestones.

Supplementary appendix 2. Format for submission of the end‑of‑the‑course project report.
