# Supplementary material for: Designing, Implementing and Optimising a Capacity‑Building Model for Infectious Disease Modelling in India
Source: Ann Glob Health. 2024 Dec 30;90(1):84. doi: 10.5334/aogh.4606 (PMC11697579; doi:10.5334/aogh.4606)
Supplement: Supplementary appendix 1. — Details about the week‑wise course topics, content, teaching–learning methods and milestones. [file agh-90-1-4606-s1.pdf]

Supplementary appendix 1: Details about the week-wise course topics, content, teaching-learning methods and milestones

Week	Topic	Content	Teaching-learning methods	Milestones
1	Introduction to Infectious Disease Epidemiology	Introduction to Infectious Disease Epidemiology<br>Basics of disease transmission and population dynamics<br>History and spread of epidemics<br>Sero-epidemiology and immunology<br>Contact studies	Online lectures<br><br>Self-reading materials	
2	Setting up Compartmental models	Models: basics and types<br>Introduction to the SIR model<br>Setting up compartmental models: difference equations<br>Setting up compartmental models: differential equations<br>Exercise 1: Setting up compartmental models and writing equations<br>Exercise 2: Calculating model parameters	Online lectures<br><br>Self-reading materials<br><br>Hands-on exercises	
3	Excel and Math basics	Excel basics<br>Math basics<br>Exercise 3: Setting up SIR and SEIR Influenza model	Recorded videos<br><br>Hands-on exercises	
4	Introducing demography and age structure into the model	Choosing the right model<br>Introducing births and deaths<br>Introducing age structure into the model<br>Case study discussion<br>Exercise 4: Changing the time step<br>Exercise 5: Building a measles model in Excel<br>Exercise 6: Introducing births and deaths into the SEIR model	Online lectures<br><br>Self-reading materials<br><br>Case studies<br><br>Hands-on exercises	Milestone 1: Submission of the first assignment by Week 4

5	R_0, herd immunity and effect of vaccination	R_0, growth rate and doubling time<br>Final epidemic size<br>Herd immunity and effect of vaccination on dynamics of infections<br>Exercise 7: Calculating Growth rate<br>Exercise 8: Comparison of predicted vs reported<br>Exercise 9: Effect of vaccination on epidemic curves	Online lectures<br><br>Self-reading materials<br><br>Hands-on exercises	
6	Effect of control measures or interventions	Effect of control measures or interventions<br>Long term dynamics of acute immunizing infections<br>Case study discussion<br>Exercise 10: Long term dynamics of measles	Online lectures<br><br>Self-reading materials<br><br>Case studies<br><br>Hands-on exercises	Milestone 2: Submission of the second assignment by Week 6
7	Stochastic models and incorporating non-random mixing	Introduction to stochastic modelling<br>How to incorporate non-random mixing?<br>Calculate R_0 if mixing is non-random<br>Age patterns in proportion of susceptible and infected population<br>Age dependency in force of infection<br>Exercise 11: Catalytic models<br>Exercise 12: Age dependent force of infection	Online lectures<br><br>Self-reading materials<br><br>Hands-on exercises	
8	Modelling other diseases: HIV, TB, Malaria, COVID-19	Modelling Sexually transmitted infections/HIV<br>Modelling malaria transmission and control<br>Infectious disease modelling during the SARS-CoV 2 pandemic<br>Modelling diseases with long incubation periods: TB	Online lectures<br><br>Self-reading materials<br><br>Case studies	Milestone 3: Submission of the third assignment by Week 8

9	Online revision and discussion forum	Online revision classes and discussion forum<br>Case studies<br>Project work	Online discussion forums<br><br>Case study discussion	
10	Online revision and discussion forum	Online revision classes and discussion forum<br>Case studies<br>Project work	Online discussion forums<br><br>Case study discussion	Milestone 4: Submission of the third assignment by Week 10
11	Online revision and discussion forum	Online revision classes and discussion forum<br>Project work	Online discussion forums	Milestone 5: Submission of the project work by Week 11
12	Contact session and exit examination	3-day face-to-face contact session and exit examination	Offline lectures<br><br>Hands-on exercises<br><br>Offline discussion forums	
