# Supplementary material for: Designing, Implementing and Optimising a Capacity‑Building Model for Infectious Disease Modelling in India
Source: Ann Glob Health. 2024 Dec 30;90(1):84. doi: 10.5334/aogh.4606 (PMC11697579; doi:10.5334/aogh.4606)
Supplement: Supplementary appendix 2. — Format for submission of the end‑of‑the‑course project report. [file agh-90-1-4606-s2.pdf]

Supplementary appendix 2: Format for submission of the end-of-the-course project report

Summative Assessment

Name:
Date of submission:

Title of the study

Background

Understanding of the disease biology

Model assumptions

Figure 1 Compartmental model for the transmission of xxxxx

List of parameters and their definitions and values

Parameter	Definition	Values	Values (in days^{-1})

Calculation of model parameters

Model equations

References
